# Recovery and Concentration of Antioxidants from Winery Wastes

**DOI:** 10.3390/molecules17033008

**Published:** 2012-03-09

**Authors:** María Luisa Soto, Enma Conde, Noelia González-López, María Jesús Conde, Andrés Moure, Jorge Sineiro, Elena Falqué, Herminia Domínguez, María José Núñez, Juan Carlos Parajó

**Affiliations:** 1 Departamento de Enxeñaría Química, Universidade de Vigo (Campus Ourense), Edificio Politécnico, As Lagoas, Ourense 32004, Spain; 2 CITI, Tecnópole, San Cibrao das Viñas, Ourense 32901, Spain; 3 Escola Técnica Superior de Enxeñaría, Universidade de Santiago de Compostela, Avda. Lope Gómez de Marzoa sn, Santiago de Compostela 15782, Spain; 4 Departamento de Química Analítica, Universidade de Vigo (Campus Ourense), Edificio Politécnico, As Lagoas, Ourense 32004, Spain

**Keywords:** winery wastes, phenolics, adsorption, resins, radical scavengers

## Abstract

: Grape and wine byproducts have been extensively studied for the recovery of phenolic compounds with antioxidant activity and a variety of biological actions. The selective recovery and concentration of the phenolic compounds from the liquid phase separated from further diluted winery wastes has been proposed. Adsorption onto non ionic polymeric resins and further desorption with ethanolic solutions was studied. Several commercial food grade resins were screened with the aim of selecting the most suited for the practical recovery of phenolic compounds with radical scavenging activity. Under the optimized desorption conditions (using Sepabeads SP207 or Diaion HP20 as adsorbents and eluting with 96% ethanol at 50 °C) a powdered yellow-light brown product with 50% phenolic content, expressed as gallic acid equivalents, was obtained. The radical scavenging capacity of one gram of product was equivalent to 2–3 g of Trolox.

## 1. Introduction

Winemaking is a seasonal activity of environmental and economic relevance in the producing countries. In some industries the final residue is the grape pomace generated in the pressing stage, but in the wine industries that produce spirits the wet distilled grape pomace is the final residue of the plant. The compounds from distilled pomace are more active than those obtained from the pressing pomace and are highly thermostable [1]. A simple alternative to recover antioxidants from distilled grape pomace, consists of the utilization of the liquid phase accompanying the pomace; this liquid presents a radical scavenging capacity comparable to synthetic antioxidants, but the phenolic purity is low (15%, dry basis) [2]. 

The phenolic compounds from the liquid phase accompanying the distilled grape pomace could be successfully adsorbed onto activated charcoal, but they could not be eluted [3]. However, the reversible adsorption of these grape phenolics onto resins was observed [4]. In that work, the liquid phase found in the distilled grape pomace was concentrated in nanofiltration membranes and further refined by adsorption onto polymeric resins and elution with ethanol. The direct adsorption onto commercial resins of the phenolic components from the winery wastes leaving the distillation stage has not been tried. 

Adsorption using nonpolar macroporous polymers presents a series of advantages, including the wide range of structures and properties available, high adsorption capacity and selectivity, good performance to recover and to separate bioactive compounds, chemical stability, relatively low cost and easy regeneration. Increasing applications of resins are found in scientific literature for the recovery and non-thermal concentration and fractionation of the crude phenolic extracts from products and byproducts of the food industry, *i.e.*, citrus peel and molasses [5], apple and grape pomace [6,7,8,9] or for solvent extracts from autohydrolysis liquors of grape pomace [10]. 

Among the phenolic compounds identified in winery products are catechins (catechin, epicatechin), flavonols (quercetin, kaempferol, myricetin), benzoic acids (gallic, protocatechuic, 4-hydroxybenzoic, syringic, gentisic) and cinnamic acids (*p*-coumaric) [7]. These dietary phenolics present high antioxidant capacity, confer protection against cronic and degenerative diseases [11], are metabolizable [12] and stable at high temperatures [1]. Based on these properties, the concentrated phenolic product from wineries could be of interest for food, pharmaceutical and cosmetic applications. Such products could be proposed as agents protecting from oxidation during storage and as bioactive components in the formulation of functional foods.

The aim of the present work was to select commercial food grade resins for the efficient adsorption of phenolic compounds with antioxidant activity present in winery wastes. Separation of solid and liquid phases from winery wastes and suitable dilution of the latter was accomplished before addressing the selective adsorption and desorption stages. Kinetic studies were carried out to compare the performance of the different resins and to establish the time required to reach equilibrium. For the resins selected on the basis of their adsorption capacity, the experimental desorption conditions were optimized to obtain concentrated extracts selectively enriched in the active components.

## 2. Results and Discussion

### 2.1. Adsorption

**Figure 1:**
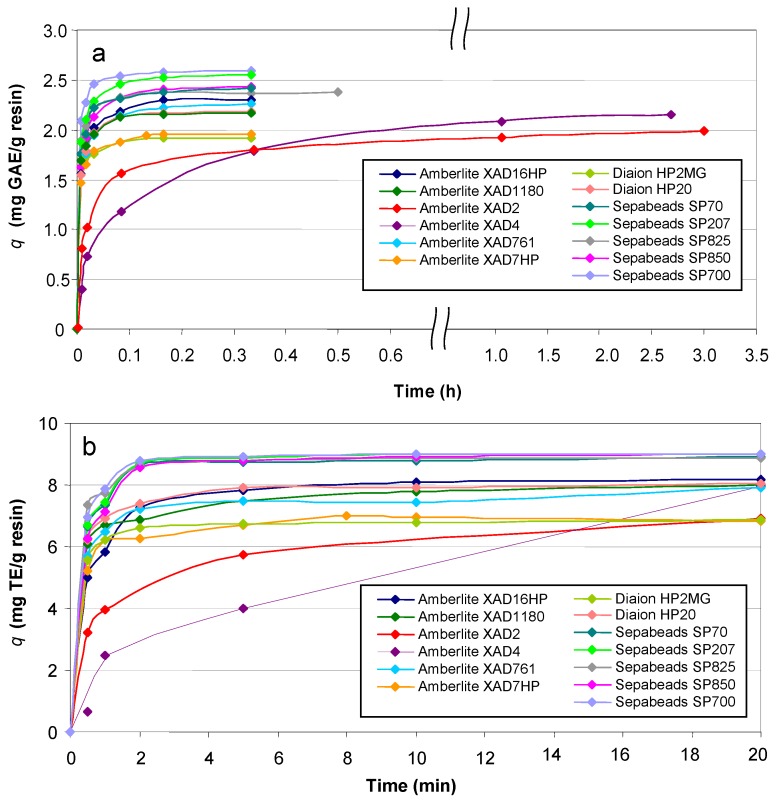
Adsorption kinetics of (**a**) phenolic compounds (expressed as gallic acid equivalents, GAE) and (**b**) ABTS radical scavengers (expressed as Trolox equivalents, TE) from winery wastes. The symbols correspond to experimental data and the lines to the calculated trend according to the selected models.

In order to select the most effective adsorbents a comparative batch adsorption experiment was performed. The total phenolics and the ABTS [2,2'-azinobis (3-ethylbenzothiazoline-6-sulfonate)] radical scavengers adsorbed were measured by *q*, as mg of gallic acid equivalent (GAE)/g resin and as mg Trolox equivalents (TE)/g resin, respectively. The kinetic data corresponding to the time course of the adsorption of phenolics and ABTS radical scavengers onto the evaluated resins are shown in Figure 1a,b. 

The adsorption yields ranged from 67 to 90%, with increasing values for Diaion HP2MG (68.7) and HP20 (75.2), Amberlite XAD2 (69.2), XAD7HP (70.4), XAD1180 (75.3), XAD761 (79.2), XAD16HP (80.2), XAD4 (81.8), and Sepabeads SP70 (83.6), SP850 (84.1), SP825 (85.6), SP207 (88.1) and SP700 (89.5). The time to reach the steady state was relatively short, and for further experiments was fixed at 0.5 h; Amberlite XAD2 and XAD4 required 3 h. These values were shorter than those needed to retain phenolics from winery wastes onto activated carbon [3]. The adsorption of ABTS radical scavengers was accomplished in shorted times than the adsorption of phenolics. In 2 min 91% of the active compounds were adsorbed onto SP207, SP70, SP825 and SP850, between 64 and 78% onto HP20, HP2MG, XAD 7HP, XAD 16HP, XAD761 and XAD1180, whereas 20 min were required to achieve these yields onto XAD2. Operating with XAD4 a different behaviour was observed, reaching 75% adsorption yield after 20 min.

The experimental data in Figure 1 were fitted to the pseudo first order model and the pseudo second order model. For all the tested resins, the *R^2^* values (0.8544–0.9855) observed with pseudo first order models were lower than those (0.9929–0.9999) found for the pseudo second order model. According to these values, summarized in Table 1, the sorption kinetics followed a pseudo second order model, also reported for other phenolics and resins [10,13,14].

**Table 1:** Regression coefficients for pseudo first and pseudo second order models for the adsorption of (**a**) total phenolics (as mg gallic acid equivalents/g resin) and (**b**) ABTS radical scavenging compounds (as mg Trolox equivalents/g resin) present in winery wastes.

*a*	**Pseudo First Order Model**				**Pseudo Second Order Model**		
	***k_1_* (min^−1^)**	***q_e_*_ calc_ (mg/g)**	**R^2^**		**k_2_ (g/mg·min)**	***q_e_*_ calc_ (mg/g)**	**R^2^**
Amberlite							
XAD2	2.74	0.80	0.8544		0.025	1.98	0.9998
XAD4	2.25	1.54	0.9376		0.011	2.18	0.9929
XAD7HP	22.5	0.49	0.9716		0.190	1.98	0.9998
XAD16HP	29.0	0.89	0.9826		0.118	2.33	0.9997
XAD761	17.9	0.70	0.9612		0.103	2.29	0.9997
XAD1180	22.8	0.47	0.9597		0.197	2.18	0.9999
Diaion							
HP20	24.5	0.56	0.9614		0.170	2.19	0.9999
HP2MG	26.8	0.38	0.9716		0.281	1.93	0.9999
Sepabeads							
SP70	16.2	0.52	0.8779		0.139	2.43	0.9998
SP207	22.3	0.65	0.9855		0.139	2.57	0.9998
SP700	26.1	0.46	0.9681		0.240	2.60	0.9999
SP825	28.6	0.55	0.9558		0.220	2.39	0.9999
SP850	20.1	0.72	0.9630		0.112	2.45	0.9998
*b*	**Pseudo First Order Model**				**Pseudo Second Order Model**		
	***k_1_* (min^−1^)**	***q_e_*_ calc_ (mg/g)**	**R^2^**		***k_2_* (g/mg·min)**	***q_e_*_ calc_ (mg/g)**	**R^2^**
Amberlite							
XAD2	5.54	3.81	0.9475		9.59	7.91	0.9998
XAD4	9.11	7.97	0.9916		2.42	8.51	0.9983
XAD7HP	30.3	1.49	0.9169		105	6.90	0.9996
XAD16HP	21.2	2.77	0.9478		29.3	8.26	0.9997
XAD761	8.63	1.41	0.5970		40.1	7.90	0.9992
XAD1180	13.5	1.80	0.9828		38.7	8.04	0.9996
Diaion							
HP20	15.7	1.33	0.7960		50.9	8.09	0.9999
HP2MG	15.7	0.80	0.7926		105	6.90	0.9999
Sepabeads							
SP70	17.1	1.23	0.6610		62.5	8.94	0.9999
SP207	43.8	2.65	0.9722		61.1	9.05	0.9999
SP700	36.0	1.84	0.9579		60.9	9.06	0.9999
SP825	28.4	1.32	0.9453		125	8.92	0.9999
SP850	19.4	1.80	0.8165		40.6	9.06	0.9999

### 2.2. Desorption

The efficient desorption of the adsorbed compounds using ethanolic solutions was proposed. Despite the fact that methanol could provide higher elution yields than ethanol [6] a food grade solvent was preferred. The process was optimized by means of a central composite design of experiments, since a systematic variation of parameters has been recommended for the recovery of purified plant extracts enriched in certain target compounds [7]. The matrix with the real values of the independent variables temperature and ethanol concentration, and the correspondent coded variables (T, E) is shown in Table 2. The experimental values for the objective functions obtained for the resins selected on the basis of their potential for selectively retaining the target compounds present in the diluted stream from winery wastes are shown in Table 2 Table 3,Table 4.

The maximal phenolics desorption yields were 67, 62 and 51%, respectively, for HP20, SP207 and XAD16HP, whereas the sugar desorption yields were 22, 31 and 19%, respectively. As a general trend, desorption of phenolic compounds is favoured by higher ethanol concentrations than desorption of sugars. The effect of temperature on the desorption yields depends on the resin and does not present a definite trend. Similar recovery yields have been reported during the alkaline elution of resins used for the simultaneous removal of phenolic compounds and polar anions from citrus peel juice and molasses [5] and for ethanol elution of phenolics from apple juice [15]. The range of phenolic content of the desorbed products was 44–51% for HP20, 35–51% for SP207 and between 30–47% for XAD16 HP. The range of sugar content of the desorbed extracts was 24–29%, for HP20, 22–31%, for SP207 and 16–26% for XAD16HP. The highest phenolic purity for HP20 was obtained for experiment 7, operating at 25 °C and eluted with 88% ethanol. An expected correlation between the phenolic content of the desorbed extracts and the radical scavenging capacity was observed. The ABTS radical scavenging capacity of the desorbed product ranged from 8.9 to 13.0 mM Trolox for HP20, from 11.2 to 13.3 mM Trolox for SP207 and from 7.6 to 14.0 mM Trolox for XAD16HP. In order to compare the efficieny of the desorbed product, these values can be referred to the activity of one gram of the dry desorbed product. These activities ranged from 1.94 to 2.63 grams of Trolox *per* gram of desorbed product for HP20, from 1.97 to 2.66 g/g for SP207 and from 1.5 to 3.1 g/g for XAD16HP. The ABTS radical scavenging capacity of one gram of BHT (butylated hydroxytoluene) was equivalent to 1.80 g Trolox, one gram of BHA (butylated hydroxyanisole) was comparable to 2.06 g Trolox and gallic acid to 4.93 g Trolox [10].

The effects of the independent variables were evaluated by a Student t-test and the significance of the models by F-test. The coefficients for the linear, quadratic and interaction effects of T and E on the objective functions are shown in Table 5 for the selected resins.

**Table 2:** Coded and real variables of a central composite design for two factors and experimental and calculated values of the objective functions during operation with Diaion HP20.

		Coded Variables			Real Variables			Objective Functions													
Exp.		T	E		T (°C)	EtOH (%)		Y_1exp_	Y_1calc_		Y_2exp_	Y_2calc_		Y_3exp_	Y_3calc_		Y_4exp_	Y_4calc_		Y_5exp_	Y_5calc_
1		−1	−1		25	48		62.0	59.6		20.9	21.3		0.444	0.439		0.242	0.255		11.4	11.1
2		1	−1		45	48		63.2	62.2		21.6	22.1		0.475	0.467		0.263	0.270		8.90	9.60
3		−1.4142	0		20.858	68		62.2	64.2		22.3	22.3		0.475	0.479		0.276	0.269		10.3	11.2
4		1.4142	0		49.142	68		63.8	63.8		23.5	23.3		0.469	0.476		0.279	0.281		11.3	10.8
5		0	−1.4142		35	40		56.7	58.7		21.6	20.9		0.434	0.441		0.268	0.255		10.7	10.3
6		0	1.4142		35	96		63. 6	63.6		21.7	22.1		0.497	0.500		0.275	0.281		12.4	13.1
7		−1	1		25	88		66.9	65.9		22.4	22.2		0.514	0.511		0.279	0.279		13.0	11.9
8		1	1		45	88		62.3	62.7		23.0	22.8		0.483	0.478		0.289	0.282		12.8	12.8
9		0	0		35	68		64.1	64.5		21.8	22.2		0.475	0.469		0.262	0.261		10.9	11.1
10		0	0		35	68		64.5	64.5		22.2	22.2		0.474	0.469		0.264	0.261		11.6	11.1
11		0	0		35	68		64.5	64.5		22.6	22.2		0.457	0.469		0.260	0.261		11.1	11.1
12		0	0		35	68		64.6	64.5		22.2	22.2		0.460	0.469		0.256	0.261		10.9	11.1
13		0	0		35	68		64.6	64.5		22.1	22.2		0.479	0.469		0.265	0.261		11.0	11.1

Y_1_: Phenolic desorption yield (%); Y_2_: Sugars desorption yield (%); Y_3_: Total phenolic content (g GAE/g extract); Y_4_: Total sugar content (g D-glucose/g extract); Y_5_: Radical scavenging activity (mM Trolox).

**Table 3:** Experimental and calculated values of the objective functions during desorption from Sepabeads SP207 resin.

Exp.		Y_1exp_	Y_1calc_	Y_2exp_	Y_2calc_	Y_3exp_	Y_3calc_	Y_4exp_	Y_4calc_	Y_5exp_	Y_5calc_
1		52.7	51.7	23.1	26.1	0.471	0.438	0.291	0.301	12.2	12.2
2		58.7	56.8	25.1	26. 8	0.454	0.423	0.272	0.275	11.7	11.2
3		55.9	56.3	24.6	22.6	0.475	0.475	0.284	0.265	12.6	12.5
4		59.5	61.2	24.6	24.5	0.488	0.482	0.268	0.260	11.4	12.0
5		49.8	51.4	31.2	28.4	0.349	0.396	0.308	0.304	11.2	11.4
6		58.3	58.9	23.2	23.9	0.511	0.458	0.285	0.262	12.4	12.7
7		58.9	58.7	21.7	22.2	0.425	0.462	0.225	0.249	12.5	12.4
8		61.6	60.5	25.1	24.2	0.446	0.487	0.251	0.267	13.3	12.7
9		59.5	57.6	25.8	24.8	0.400	0.400	0.244	0.240	11.6	11.7
10		58.3	57.6	24.4	24.8	0.423	0.400	0.254	0.240	11.2	11.7
11		58.2	57.6	24.6	24.8	0.401	0.400	0.234	0.240	11.8	11.7
12		55.9	57.6	25.2	24.8	0.372	0.400	0.236	0.240	12.0	11.7
13		56.1	57.6	23.9	24.8	0.405	0.400	0.233	0.240	11.9	11.7

Y_1_: Phenolic desorption yield (%); Y_2_: Sugars desorption yield (%); Y_3_: Total phenolic content (g GAE/g extract); Y_4_: Total sugar content (g D-glucose/g extract); Y_5_: Radical scavenging activity (mM Trolox).

**Table 4:** Experimental and calculated values of the objective functions during desorption from Amberlite XAD16HP resin.

Exp.			Y_1exp_	Y_1calc_	Y_2exp_	Y_2calc_	Y_3exp_	Y_3calc_	Y_4exp_	Y_4calc_	Y_5exp_	Y_5calc_
1			41.7	42.1	16.6	16.6	0.350	0.387	0.220	0.245	7.98	7.65
2			46.1	46.0	16.5	17.2	0.307	0.328	0.176	0.199	8.87	8.87
3			49.3	47.8	18.5	18.1	0.413	0.392	0.249	0.238	8.43	8.70
4			50.5	49.8	19.1	18.1	0.397	0.399	0.241	0.235	9.22	9.02
5			39.5	39.8	16.1	15.6	0.356	0.319	0.233	0.202	7.63	7.85
6			45.1	42.7	15.5	14.7	0.297	0.315	0.165	0.177	8.92	8.77
7			44.3	46.6	16.0	16.6	0.323	0.321	0.188	0.183	9.36	9.29
8			43.7	45.5	15.1	16.1	0.408	0.390	0.234	0.225	8.25	8.52
9			51.5	50.4	18.4	17.8	0.410	0.437	0.235	0.247	13.3	13.3
10			50.9	50.4	17.8	17.8	0.419	0.437	0.235	0.247	14.0	13.3
11			49.0	50.4	16.5	17.8	0.469	0.437	0.254	0.247	12.9	13.3
12			50.5	50.4	18.8	17.8	0.432	0.437	0.259	0.247	13.1	13.3
13			50.0	50.4	17.5	17.8	0.452	0.437	0.235	0.247	13.4	13.3

Y_1_: Phenolic desorption yield (%); Y_2_: Sugars desorption yield (%); Y_3_: Total phenolic content (g GAE/g extract); Y_4_: Total sugar content (g D-glucose/g extract); Y_5_: Radical scavenging activity (mM Trolox).

**Table 5:** Regression coefficients and statistical parameters for the objective functions*.*

		Y_1_: Phenolic Desorption Yield (%)				Y_2_: Sugars Desorption Yield (%)			Y_3_: Total phenolic Content(g GAE/g extract)				Y_4_: Total Sugar Content (g D-glucose/g extract)			Y_5_: Radical Scavenging Activity (mM Trolox)			
		coefficient		probability		coefficient	probability		coefficient	probability			coefficient	probability		coefficient		probability	
***Diaion HP20***																			
**a_0_**		64.5		3.80 × 10^−12^		22.2	1.64 × 10^−12^		0.469		1.32 × 10^−12^		0.261	5.22 × 10^−11^			11.1	5.14 × 10^−9^	
**a_T_**		−0.142		0.799		0.362	0.063		−0.001		0.751		0.004	0.212			−0.158	0.561	
**a_E_**		1.70		0.016		0.393	0.048		0.021		0.000		0.009	0.023			0.989	0.007	
**a_TE_**		−1.42		0.103		−0.043	0.857		−0.015		0.015		−0.003	0.547			0.587	0.154	
**a_TT_**		−0.228		0.704		0.298	0.133		0.004		0.292		0.007	0.082			−0.057	0.844	
**a_EE_**		−1.66		0.023		−0.333	0.099		0.001		0.797		0.004	0.325			0.302	0.314	
**R^2^**		0.758				0.721			0.878				0.688				0.728		
**Error **		1.519				0.463			0.009				0.009				0.734		
**F **		4.39				3.62			10.1				3.09				3.75		
***Sepabeads SP207***																			
**a_0_**			57.6	2.19 × 10^−11^		24.8	1.86 × 10^−8^		0.400		9.74 × 10^−8^		0.240	1.14 × 10^−8^			11.7	1.03 × 10^−10^	
**a_T_**			1.72	0.027		0.672	0.367		0.002		0.864		−0.002	0.767			−0.184	0.277	
**a_E_**			2.65	0.004		−1.60	0.056		0.022		0.169		−0.015	0.051			0.447	0.024	
**a_TE_**			−0.819	0.379		0.348	0.735		0.009		0.647		0.011	0.248			0.347	0.161	
**a_TT_**			0.576	0.413		−0.621	0.433		0.039		0.039		0.011	0.143			0.266	0.157	
**a_EE_**			−1.25	0.101		0.673	0.397		0.013		0.420		0.022	0.015			0.166	0.355	
**R^2^**			0.821			0.535			0.572				0.730				0.684		
**Error **			1.74			1.97			0.0405				0.0178				0.442		
**F **			6.41			1.61			1.87				3.79				3.03		
***Amberlite XAD16HP***																			
**a_0_**		50.4		4.94 × 10^−11^		17.8	1.47 × 10^−9^		0.437	8.12 × 10^−9^			0.247	2.70 × 10^−8^		13.3		1.92 × 10^−11^	
**a_T_**		0.694		0.290		0.021	0.953		0.002	0.837			−0.001	0.879		0.114		0.443	
**a_E_**		1.02		0.137		−0.289	0.433		−0.001	0.919			−0.009	0.266		0.323		0.0545	
**a_TE_**		−1.27		0.182		−0.283	0.583		0.032	0.075			0.022	0.069		−0.500		0.039	
**a_TT_**		−0.787		0.265		0.173	0.656		−0.020	0.125			−0.006	0.510		−2.24		1.45 10^−6^	
**a_EE_**		−4.58		0.0002		−1.32	0.009		−0.059	0.001			−0.029	0.008		−2.52		6.62 10^−7^	
**R^2^**		0.889				0.672			0.819				0.736			0.985			
**Error **		1.71				0.983			0.031				0.021			0.396			
**F **		11.2				2.87			6.35				3.90			91.6			

The quadratic effect of the ethanol concentration in the eluting solvent was only significant on desorption yields whereas the linear effect of this variable was significant on all objective functions during operation with HP20. With SP207, the linear effect of the ethanol concentration was significant on all functions, except on the phenolic content of the product. Operating with XAD16HP the quadratic effect of the ethanol concentration was the most significant, followed by the interaction effect between temperature and ethanol, particularly on the phenolic and sugar content of the extracts and on the radical scavenging capacity. 

**Figure 2:**
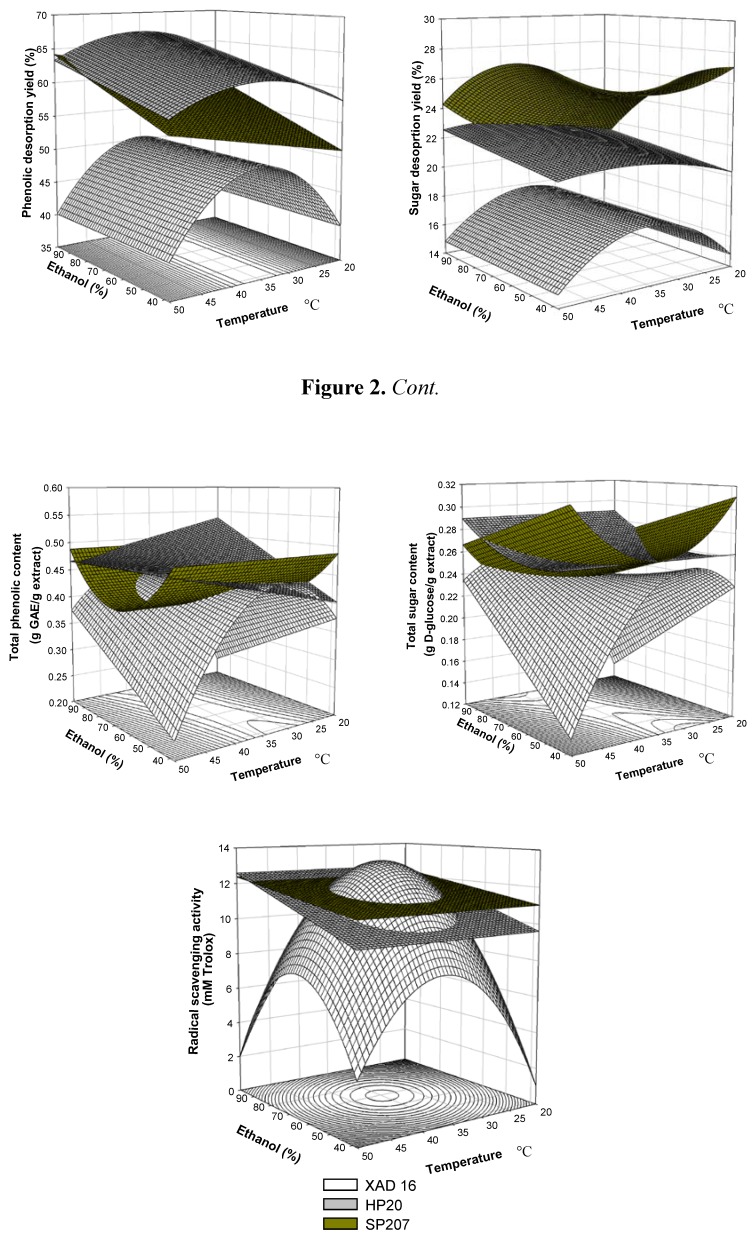
Response surface of the objective functions studied to optimize the desorption stage of phenolic compounds, sugars and radical scavengers from XAD-16HP, SP207 and HP20 resins.

A comparison between the experimental and calculated objective functions for randomly selected operational conditions confirmed the good prediction ability of the models for all resins (Table 6). The response surface models and the contour plots defined with the variables significant at 90% level are shown in Figure 2 for the studied objective functions.

**Table 6:** Comparison between experimental and calculated values of the objective functions at the optimal conditions.

		Real Variables			Coded Variables			Objective Functions													
Resin		T (°C)	EtOH (%)		T	E		Y_1exp_	Y_1calc_		Y_2exp_	Y_2calc_		Y_3exp_	Y_2calc_		Y_4exp_	Y_2calc_		Y_5exp_	Y_5calc_
HP20		50	96		1.5	1.4142		56.8	59.8		22.9	23.2		0.473	0.475		0.266	0.297		13.3	14.0
SP207		50	96		1.5	1.4142		60.0	61.0		21.6	24.2		0.521	0.570		0.291	0.318		12.7	13.7
XAD16HP		45	96		1	1.4142		39.3	40.8		16.3	14.5		0.272	0.343		0.163	0.202		8.39	5.93

Y_1_: Phenolic desorption yield (%); Y_2_: Sugars desorption yield (%); Y_3_: Total phenolic content (g GAE/g extract); Y_4_: Total sugar content (g D-glucose/g extract); Y_5_: Radical scavenging activity (mM Trolox).

The eluted product presented a yellow-light brown colour, powder texture and a characteristic winery odour with a phenolic content of 50% d.b., higher than that of some commercial ingredients [16] and other concentrated refined products, such as those from olive mill wastewaters concentrated with resins [17]. The final extracts contained 25% sugars and for some applications the removal of organic acids and sugars would be desirable [8].

The phenolic compounds were expressed as total phenolics, although the liquid chromatography profile of the product obtained with the selected resins (Sepabeads SP207) revealed the presence of monomeric compounds (gallic acid, catechin, epicatechin, quercetin), oligomeric and polymeric phenols. The degree of polymerization of the phenolic compounds recovered from the winery wastes was 3, slightly higher than those from distillery byproducts of the same origin and different year obtained in the retentates of nanofiltration membranes and further recovered with Sepabeads SP700 [4]. Degrees of polymerization in the range 1–3.7 have been reported for other grape extracts, and this criteria was confirmed to be important for modulating the antioxidant capacity with the lowest cytotoxic efects [18,19]. The compounds found in the dichloromethane soluble fraction are shown in Table 7, both for the liquid phase separated from the winery wastes and further diluted and for the product desorbed from selected polymeric resins under optimal conditions. Most components were not detected in the liquid phase separated from winery wastes further diluted, but were found in the concentrated desorbed product. The most abundant were the monoethyl succinate, propanoic acid and benzeneacetaldehyde, followed by two acids (acetic and lauric), two alcohols (2-methyl-2-butanol and 2-phenylethanol) and two esters (1,3-propanediol acetate and ethyl lactate). Most of these compounds are generated during the alcoholic fermentation at higher contents and therefore, they can be found in winery wastes.

**Table 7:** Composition of the DCM soluble fractions from the liquid phase separated from winery wastes further diluted (A) and from the product desorbed from Sepabeads SP207 with 96% ethanol at 50 °C (B), analysed by GC-MS.

t (min)	Name	Relative Area to 3-Octanol	
		A	B
8.57	2-methyl-2-butanol	5.979	7.195
10.14	1,1-diethoxy-3-methylbutane	ND	0.326
10.75	2-methylpropanol (isobutanol)	ND	0.113
16.73	3-octanone	0.776	0.191
18.09	3-hydroxy-2-butanone (acetoin)	ND	1.015
19.39	methyl lactate	ND	0.263
20.40	ethyl lactate	ND	4.559
21.20	2-hydroxy-2-methyl-4-pentanone	0.272	ND
21.69	3-ethoxy-1-propanol	ND	0.232
25.12	acetic acid	0.180	7.900
28.07	benzaldehyde	ND	0.692
28.58	propanoic acid	ND	15.648
29.91	2,3-butanediol	ND	0.689
31.93	methyl benzoate	ND	1.299
32.27	dihydro-2(3H)-furanone (γ-butyrolactone)	ND	1.453
32.78	benzeneacetaldehyde	ND	13.022
33.74	3-methylbutanoic acid (isovaleric acid)	ND	0.206
33.90	diethyl succinate	ND	0.212
35.48	(2,2 diethoxyethyl)benzene	ND	0.294
36.21	1,3-propanediol diacetate	ND	5.869
37.84	1,3-propanediol	ND	0.203
38.65	ethyl propanoate	ND	1.108
40.18	hexanoic acid	ND	0.371
40.63	*N*-(3-methylbutyl)acetamide	ND	0.844
42.44	phenylethanol	ND	3.434
54.81	2-ethylhexyl-2-hydroxybenzoate	ND	0.543
57.46	monoethyl succinate	ND	50.183
60.22	dodecanoic acid (lauric acid)	ND	5.589
62.32	diethyl succinate	ND	0.683
72.51	homovanillyl alcohol (4-hydroxy-3-methoxyphenylethyl alcohol)	ND	0.804

ND, non detected.

## 3. Experimental

### 3.1. Materials

*Winery wastes*. Winery wastes from *Cooperativa Vitivinícola do Ribeiro* (Ribadavia, Ourense, Spain), years 2007–2008, were collected and processed to separate the solid and liquid fractions. The obtained liquid phase was centrifuged to remove suspended solids, diluted with tap water and stored at 4 °C until use. The total phenolic and sugar content in the diluted liquid phase were 1.8 g (expressed as gallic acid equivalents)/L and 6.0 g/L, respectively.

*Resins.* The food grade resins used were an acrylic polymer, Amberlite XAD7HP, two resins with a formaldehyde-phenol polycondensed matrix, Amberlite XAD761 and Amberlite XAD1180, and three PS-DVB resins, Amberlite XAD2, Amberlite XAD4 and Amberlite XAD16, supplied by Sigma Chemical Corporation. PS-DVB copolymers with different hydrophobicity, Sepabeads SP700, Sepabeads SP207, Sepabeads SP825, Sepabeads SP850 and Diaion HP20, a resin with a polymethacrylate estructure, Diaion HP2MG and a chemically modified PS-DVB polymer, Sepabeads SP70, were kindly supplied by Resindion S.R.L. (Mitsubishi Chemical Corporation). The physicochemical characteristics of these resins are summarized in Table 8.

**Table 8:** Physicochemical characteristics of the commercial resins used for the recovery of phenolic compounds from winery wastes.

Resin Name	Structure	Surface Area (m^2^/g)	Pore Radius (Å)	Porosity (mL/g)	Particle Size (mm)	Density (g/mL)
**Amberlite**						
XAD2	PS-DVB	330	90	0.65	0.25–0.84	1.02
XAD4	PS-DVB	725	40	0.98	0.25–0.84	1.02
XAD7HP	Acrylic ester	450	90	1.14	0.25–0.84	1.05
XAD16	PS-DVB	800	100	1.82	0.25–0.84	1.02
XAD761	Phenol-formaldehyde	300	600	0.43	0.56–0.76	1.11
XAD1180	Phenol-formaldehyde	600	300	1.68	0.35–0.60	1.01
**Diaion**						
HP20	PS-DVB	600	260	1.3	0.25–0.60	1.01
HP2MG	Polymethacrylate	470	170	1.2	0.25–0.60	1.09
**Sepabeads**						
SP70	Chemically modified PS-DVB (Br-PS-DVB)	800	70	1.6	0.25–0.85	1.01
SP207	PS-DVB	630	120	1.1	0.25–0.60	1.18
SP700	PS-DVB	1200	90	2.3	0.25–0.70	1.01
SP825	PS-DVB	1000	57	1.4	0.30–0.50	1.01
SP850	PS-DVB	1000	38	1.2	0.30–0.80	1.01

The resins were activated by contact with sufficient methanol to cover the resin bed by 2.5–5 cm (Sigma-Aldrich, Madrid, Spain). Resins and methanol were blended gently by shaking one minute and then the suspension was stirred at 175 rpm and 25 °C during 15 min. Before use resins were rinsed with deionized water at a liquid to solid ratio of 5 (g/g). The moisture content of the resins was determined by drying the beads in an oven at 100 °C up to constant weight, and adsorption experiments were carried out utilizing known amounts of resins. 

*Absorption.* The centrifuged winery liquid wastes were contacted in batch mode with weighed quantities of hydrated resins in sealed Erlenmeyer flasks at 25 °C in an orbital shaker at 175 rpm. The concentration of phenolics adsorbed at time *t* onto a mass unit of resin (*q*_t_, mg/g) was measured as gallic acid equivalents and calculated by the equation:



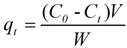
(1)


where *C_0_* and *C_t_* are the concentrations of phenol in the aqueous solution (mg/L) at the initial stage and at *t* time, respectively, *V* is the volume of the solution added into the flask (L), and *W* is the weight of the wet resin (g). Experiments were performed in triplicate.

The kinetic assays were performed in 25 mL Erlenmeyer flasks, with 5 mL of the liquid phase separated from winery wastes and further diluted and 3 g of resins at an initial pH 4.0, at 25 °C for up to 3 h. The content of each flask was filtered trough a 0.45 μm membrane filter and the liquid phase was analyzed. The pseudo-first-order rate equation of Lagergren is generally described by equation (2), and assumes that the rate of solute uptake is proportional to the gradient in saturation concentration.



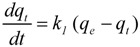
(2)


where *q_t_* and *q_e_* are the amount of phenol adsorbed (mg/g) at contact time *t* (min) and at equilibrium, *k_1_* is the pseudo-first-order rate constant (min^−1^). Integration and linearization leads to:



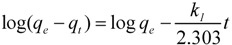
(3)


The pseudo-second order kinetic model is represented by: 



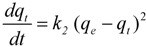
(4)


where t is the contact time, *q*_t_ and *q*_e_ are the concentrations of phenolics adsorbed (expressed as mg/g) at the considered time and at the equilibrium respectively, *k*_2_ is the pseudo-second order kinetic parameter. Integration and linearization results in:



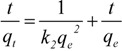
(5)


*Washing*. Distilled water was used to remove unadsorbed compounds susceptible of reducing purity of the extracts desorbed in further stages. Washing was performed in two stages with distilled water at a water:resin ratio of 3 (g:g) in an orbital shaker (175 rpm) at 25 °C for 20 min.

*Desorption.* Ethanol/water mixtures were selected on the basis of availability, suitability for food uses, and the reported cleanup capability. Optimization of the desorption conditions to produce a purified antioxidant extract was addressed by applying a central composite factorial design. The independent variables were temperature (°C), and the ethanol content (%, v/v), coded as: 



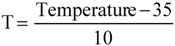
(6)




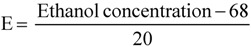
(7)


The dependent variables or objective functions were expressed according to the general expression of a second order polynomial equation (equation 8). The phenolic desorption yield (Y_1_, %), the sugars desorption yield (Y_2_, %), the total phenolic content (Y_3_, g GAE/g extract), the total sugar content (Y_4_, g D-glucose/g extract) and the radical scavenging activity (Y_5_, mM Trolox) were expressed as a function of linear, interaction, and second-order terms involving the normalized, dimensionless variables T and E:



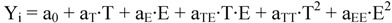
(8)


where Y_i(i = 1–5)_ are the dependent variables, T and E are the dimensionless, normalized, independent variables, and a_0_, a_T_, a_E_, a_TE_, a_TT_ and a_EE_ are regression coefficientes calculated from experimental data.

During elution the resin, saturated at conditions previously selected, was contacted with the ethanol:water solution at a moist resin to ethanol ratio 1:3 (g:mL) (resin moisture is approximately 65%). The desorbed extract was analyzed for phenolic and sugar content and for radical scavenging activity. The resin regeneration procedure consisted on leaving the resin overnight in 1 M NaOH and further washing with deionized water.

### 3.2. Analytical Methods

The total phenolic content was determined by the Folin-Ciocalteu assay [20], and expressed as Gallic Acid Equivalents (GAE). The total sugar content was determined by the Antrone method [21], and expressed as Glucose Equivalents.

The phenolic compounds were analysed in an Agilent HPLC 1100 equipped with a Waters Spherisorb ODS-2 column (5 μm, 250 mm × 4.6 mm) and DAD detector, operating at 30 °C with a flow rate of 1 mL/min. Gradient elution using solvent A (acetonitrile/water/formic acid, 10:85:5) and solvent B (acetonitrile/water/formic acid, 90:5:5) was performed: 0 min, 100% A, 0% B; 40 min, 85% A, 15% B; 45 min, 0% A, 100% B; 60 min, 100% A, 0% B. Quantification was performed from calibration curves obtained with standard compounds diluted in methanol. 

Samples were conditioned for gas chromatography-mass spectrometry analysis (GC-MS). About 0.25 mL of 3-octanol (10 ppm) was added as internal standard into 25 mL of a diluted extract solution (0.5 g extract/L). This mixture was extracted with dichloromethane (DCM). The organic phase was transferred to a graduate glass tube and concentrated under nitrogen. GC-MS analysis was carried out in splitless mode in a Hewlett-Packard 5890-II gas chromatograph coupled to a mass spectrometer HP-5970 using He as carrier gas. Separation was performed using a 60 m × 0.25 mm × 0.25 μm film thickness HP-Innowax capillary column. The temperature was maintained at 45 °C for 1 min, increased to 230 °C at 3 °C/min, and then held for 30 min. Mass spectrometer was in EI mode (electron energy 70 eV; source temperature 250 °C), and data acquisition was made in scanning mode from 30 to 300 amu/s and 1.9 spectra/s. Compounds were identified by comparison of the retention time and mass spectra with library data of mass spectra (Wiley 7n) and authentic compounds. Quantification was performed by the internal standard method (using 3-octanol as standard).

The degree of polymerization of the procyanidins was estimated by RP-HPLC analysis of the depolymerized fractions present in the reaction media after the thiolysis at 65 °C of the desorbed product diluted in methanol [18]. RP-HPLC analysis were carried out in a Smart (Amersham-Pharmacia Biotech, Uppsala, Sweden) equipment fitted with a C18 Hypersil ODS column (Supelco). Elution was carried out at a flow rate of 1 mL/min of solvent A (0.1% aqueous TFA) and solvent B (0.082% TFA in water/CH_3_CN (1:4)). The gradient, expressed as concentration of B varied as follows: 0–30 min from 12% to 30%, 30–40 min from 30% to 100%, 40–45 min from 100% to 12%.

The antioxidant activity was evaluated as the radical scavenging using the *TEAC (Trolox Equivalent Antioxidant Capacity)* assay. A 7 mM ABTS [2,2'-azinobis (3-ethyl-benzothiazoline-6-sulfonate)] stock solution was reacted with 2.45 mM potassium persulfate and kept in the dark at room temperature for 12–16 h before use. The formed ABTS^•+^ solution was diluted with phosphate buffer saline (PBS) (pH 7.4) to an absorbance of 0.700 at 734 nm and equilibrated at 30 °C. One mL of diluted ABTS^•+^ solution was mixed with 10 μL of antioxidant compounds or Trolox standards in ethanol or PBS and the absorbance was read up to 6 min, using appropriate solvent blanks. The percentage inhibition of absorbance at 734 nm was calculated as a function of the concentration of extracts and Trolox.

## 4. Conclusions

Commercial polymeric resins have been proposed to recover and concentrate the phenolic compounds with radical scavenging activity present in winery wastes. The adsorption of both total phenolics and the ABTS radical scavengers followed a pseudosecond order model. During desorption, the ethanol concentration of the eluting solvent was the most influencing variable. The eluted concentrated powder product was light in colour and contained 50% phenolics and 25% sugars. Phenolic acids, the most abundant being gallic acid, flavonoids, such as catechin, epicatechin and quercetin, and oligomeric fractions were detected by liquid chromatography. The results from the present study confirmed the potential of commercial resins to selectively recover and concentrate the phenolic compounds present in the winery wastes, after separation of liquid and solid phases and appropriate dilution of the liquid.
